# Antioxidant and Antidiabetic Effects of Flavonoids: A Structure-Activity Relationship Based Study

**DOI:** 10.1155/2017/8386065

**Published:** 2017-11-28

**Authors:** Murni Nazira Sarian, Qamar Uddin Ahmed, Siti Zaiton Mat So'ad, Alhassan Muhammad Alhassan, Suganya Murugesu, Vikneswari Perumal, Sharifah Nurul Akilah Syed Mohamad, Alfi Khatib, Jalifah Latip

**Affiliations:** ^1^Department of Pharmaceutical Chemistry, Faculty of Pharmacy, International Islamic University Malaysia (IIUM), 25200 Kuantan, Pahang, Malaysia; ^2^School of Chemical Sciences and Food Technology, Faculty of Science and Technology, Universiti Kebangsaan Malaysia (UKM), 46300 Bangi, Selangor, Malaysia

## Abstract

The best described pharmacological property of flavonoids is their capacity to act as potent antioxidant that has been reported to play an important role in the alleviation of diabetes mellitus. Flavonoids biochemical properties are structure dependent; however, they are yet to be thoroughly understood. Hence, the main aim of this work was to investigate the antioxidant and antidiabetic properties of some structurally related flavonoids to identify key positions responsible, their correlation, and the effect of methylation and acetylation on the same properties. Antioxidant potential was evaluated through dot blot, 2,2-diphenyl-1-picrylhydrazyl (DPPH) radical scavenging, ABTS^+^ radical scavenging, ferric reducing antioxidant power (FRAP), and xanthine oxidase inhibitory (XOI) assays. Antidiabetic effect was investigated through *α*-glucosidase and dipeptidyl peptidase-4 (DPP-4) assays. Results showed that the total number and the configuration of hydroxyl groups played an important role in regulating antioxidant and antidiabetic properties in scavenging DPPH radical, ABTS^+^ radical, and FRAP assays and improved both *α*-glucosidase and DPP-4 activities. Presence of C-2-C-3 double bond and C-4 ketonic group are two essential structural features in the bioactivity of flavonoids especially for antidiabetic property. Methylation and acetylation of hydroxyl groups were found to diminish the* in vitro* antioxidant and antidiabetic properties of the flavonoids.

## 1. Introduction

**Figure 1:**
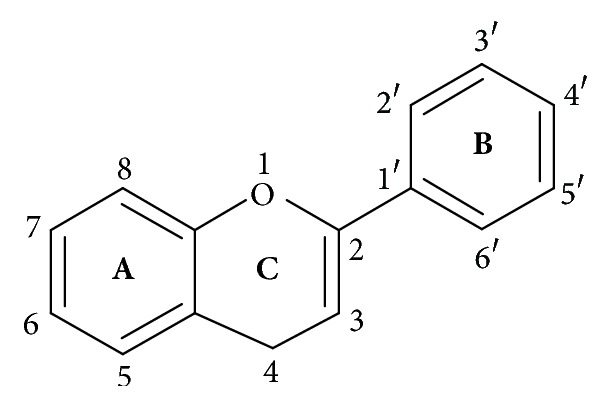
Basic structure of flavonoids consists of a fused A and C rings, with phenyl B ring attached through its 1′ position to the 2 position of the C ring.

Flavonoids, a group of hydroxylated phenolic substances known to be potent free radical scavengers, have attracted a tremendous interest as possible therapeutics against free radical mediated diseases, particularly diabetes mellitus [1–3]. Flavonoids are benzo-*γ*-pyrone derivatives and are known to be synthesized by plants in response to microbial infection. They (Figure 1) are classified according to their side group positions and substitutions. The chemical nature of these polyphenolic substances depends on their structural class, degree of hydroxylation, other substitutions and conjugations, and degree of polymerization. Their different pharmacological effects are mostly structure dependent. The protective effects of flavonoids in biological systems are ascribed to their capacity to transfer hydrogen or electrons free radical [4], activate antioxidant enzymes [5], chelate metal catalyst [6], reduce *α*-tocopherol radicals [7], and inhibit oxidases [8].

**Figure 2:**
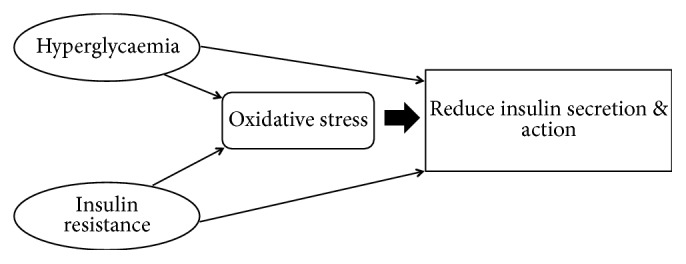
Relationship of hyperglycaemia, insulin resistance, and oxidative stress.

Reactive oxygen species (ROS) are capable of oxidizing cellular proteins, nucleic acids, and lipids. Studies and clinical evidences have shown that the generation of ROS increases in both types of diabetes and that the onset of diabetes is closely associated with oxidative stress mainly through oxidation, nonenzymatic protein glycation, and oxidative degradation of glycated proteins [9, 10]. Elevation of ROS such as mitochondrial superoxide in endothelial cell [11] and endoplasmic reticulum stress [8] followed by reduced antioxidant defense mechanism provokes cellular and enzyme damage, and lipid peroxidation which subsequently lead to the development and progression of insulin resistance and hyperglycaemia [12]. Both hyperglycaemia and insulin resistance are linked to the generation of an oxidative stress, which can also produce an impaired insulin action (Figure 2). It has been shown that hyperlipidemia acts to generate oxidative stress in the mitochondria through the same pathway as hyperglycemia [13]. Studies have also shown that antioxidants are able to improve insulin action [14–16]. Since the early 1980s, the potential effects of flavonoids in diabetes mellitus have been studied eminently for type 2 compared to that for type 1 [13, 17]. Flavonoids showing potent antioxidant activity have been suggested to be beneficial in the management of diabetes mellitus. The ability of antioxidants to protect against the deleterious effects of hyperglycemia and also to enhance glucose metabolism and uptake should be considered as a lead alternative in diabetes mellitus treatment. On top of their antioxidative effect, flavonoids may act on biological targets involved in type 2 diabetes mellitus such as *α*-glycosidase and DPP-4. Being a radical scavenger, flavonoids can effectively prevent and/or manage type 2 diabetes mellitus. Since flavonoids are directly associated with human dietary ingredients and health, there is a need to evaluate structure activity relationship with regard to understanding their functions more accurately.

Several traditional medicinal plants rich in flavonoid contents have been reported to exert antioxidant and antidiabetic effects [18–20]. In this regard,* Tetracera indica* Merr. and* Tetracera scandens* (L.) Merr. (family Dilleniaceae) have been reported to contain rich amount of flavonoids [8, 21–23].* T. indica* commonly known as akar mempelas paya and* T. scandens* commonly known as mempelas kasar are traditionally used to manage diabetes mellitus in different parts of Malaysia [21–24]. In this research work, different bioassays were applied to evaluate the antioxidant and antidiabetic activities of flavonoids isolated from* T. indica *and* T. scandens* and their semisynthetic and structural analogs. Since these compounds are based on the flavonoids molecules, configuration and type of substitution may influence the antioxidant and antidiabetic activities. Hence, this study was aimed at investigating the role of hydroxyl, methoxy, and acetate groups in flavonoids structure owing to the fact that the antioxidant and antidiabetic potentials of flavonoids are affected by the presence of different functionalities about their nuclear structure. Therefore, attempts were made to investigate their structures' relationship and correlation for antioxidant and antidiabetic effects. Further advancement of this research work may lead to the development of nutritional product and semisynthetic analogs that retain substantial antidiabetic capacity with minimal adverse effects.

## 2. Materials and Methods

### 2.1. Chemicals, Reagents, and Solvents

8-Hydroxy-7-methoxyflavone, (+)-catechin, (−)-epicatechin, quercetin (control), ascorbic acid, trolox, ABTS+ radical, potassium persulphate, xanthine, xanthine oxidase, anhydrous potassium carbonate, anhydrous sodium sulphate, acetic anhydride, pyridine, and 2,2-diphenyl-1-picrylhydrazyl (DPPH) were purchased from Sigma-Aldrich (Singapore). 2,4,6-Tris(2-pyridyl)-s-triazine (TPTZ, 99%), iron (III) chloride hexahydrate, and sodium acetate were purchased from Sigma-Aldrich (St. Louis, MO, USA). Allopurinol was purchased from Nacalai Tesque (Japan). Dipeptidyl peptidase-4 (DPP-4) inhibitor assay kit was purchased from Cayman (Michigan, USA). *α*-Glucosidase from* Saccharomyces cerevisiae* was purchased from Megazyme (Ireland). Methanol, chloroform, ethyl acetate, acetone, ethanol, dimethyl sulphoxide (DMSO), dimethyl sulphate, and thin layer chromatography (TLC) plates were purchased from Merck (Germany).

### 2.2. Collection and Preparation of Plant Material

Fresh leaves (10 kg) of* T. indica* and* T. scandens* each were collected from the local garden Taman Pertanian, Indera Mahkota, 25200 Kuantan, Pahang, Malaysia. Identification of the plants was performed by the taxonomists of Taman Pertanian and Kulliyyah of Pharmacy, IIUM. Later, the samples of both plants were deposited in the herbarium of Kulliyyah of Pharmacy, IIUM, Kuantan, to get their voucher specimen numbers (NMPC-QSTI39 and NMPC-QU24) for the future references. The same plant materials were compared with the already deposited specimens of the same plants in the herbarium of Kulliyyah of Pharmacy, IIUM. 5 kg of powdered material of each plant's leaves was macerated in 20 L analytical grade distilled MeOH for 24 h at room temperature in the dark, filtered, and concentrated in a reduced pressure using Buchi rotary evaporator. Recovered MeOH was again poured into the already extracted powdered material, filtered, and concentrated to remove the entire solvent. The whole process was repeated about four times till the plant material stopped giving coloration as well as to ensure maximum yield of methanol soluble (bioactive) compounds from the plants material. The concentrated extracts free of methanol were further subjected to freeze-drying process to remove water content from the resultant extracts to make them completely dried. Finally, MeOH extracts of the leaves of* T. indica *(250 g) and* T. scandens *(180 g) were successfully obtained.

### 2.3. Fractionation of* T. indica* and* T. scandens* Leaves MeOH Extracts and Flavonoids Isolation

Conventional maceration, solvent extraction, silica gel, and sephadex LH_20_ column chromatographies methods were effectively used to isolate some desirable flavonoids from the leaves MeOH extracts of* T. indica *and* T. scandens, *respectively. Initially, flavonoids were isolated from the leaves MeOH extract of* T. indica *as has been briefly described below. Fractionation of the leaves MeOH extract was done using separatory funnel with regard to removing undesirable substances initially. Typically, methanol extract (250 g) was dissolved in distilled water and treated with hexane until the hexane portion became clearly separated or visible. Collected hexane portion was recovered through rotary evaporator. The combined hexane fraction (15.7 g) was considered as nonpolar extract (hexane soluble compounds) of the leaves of* T. indica*. Secondly, the remaining hexane insoluble portion was treated with ethyl acetate (EtOAc) in the same manner. After concentrating through rotary evaporator, the collected fraction was considered as EtOAc fraction (85.45 g) of the leaves methanol extract of* T. indica*. Finally, ethyl acetate insoluble part was treated with butanol by following the same procedure to get butanol fraction (40.25 g) of the leaves methanol extract of* T. indica*. Initially, isolation of flavonoids from EtOAc fraction was performed by silica gel 60 (63–200 *μ*m) column chromatography followed by small preparative columns containing silica gel 60 (63–200 *μ*m) and sephadex LH 20. Ethyl acetate fraction of* T. indica* leaves MeOH extract using repeated silica gel and sephadex LH 20 column chromatographies afforded three different flavones, namely, wogonin, norwogonin, and techtochrysin. Similar aforesaid method was strictly followed to isolate some desirable already reported flavonoids from the* T. scandens* leaves MeOH extract that afforded two flavones (hypolaetin and isoscutellarein) and two flavonols (kaempferol and quercetin) after repeated silica gel and sephadex LH_20_ column chromatographies and recrystallization techniques [24]. These compounds structures were characterized by spectroscopic analysis (NMR, IR, UV, and mass spectrometry). Their spectral data were further compared with the previously reported spectral data of the similar compounds already isolated from different plants to ensure their correct structures.

### 2.4. Semisynthetic Analog

#### 2.4.1. Methylation of Wogonin

Wogonin (100 mg) was methylated with dimethyl sulphate and anhydrous potassium carbonate in dry acetone on water bath for 3 h; upon completing the reaction, product was filtered, dried, and treated with chloroform. Chloroform soluble fraction was extracted with water and dried over anhydrous sodium sulphate; then it was put for crystallization with ethanol to get pure form of methyl ether of wogonin as needle shaped yellow crystals [25].

#### 2.4.2. Acetylation of Wogonin and Norwogonin

Wogonin and norwogonin (100 mg) were separately acetylated with pyridine (5 mL) and acetic anhydride (15 mL) on the water bath for at least 4 h. Upon completing the reaction, mixture was poured slowly into crushed ice with continuous stirring. After some time, the precipitation was dissolved in chloroform and crystallized from ethanol as white needle shaped crystals [25].

### 2.5. Rapid Screening of Radical Scavenging Activity by Dot Blot Assay

An aliquot (20 *μ*L) of each dilution of each group was carefully loaded on a 10 × 10 cm^−1^ TLC plate and allowed to dry for 10 min. Drops of each sample were loaded in the order of decreased concentration along the 16 columns. The staining of the silica plate was based on the procedure of Soler-Rivas et al. [26]. The sheet bearing the dry spots was placed upside down for 10 s in a 0.4 mM DPPH solution in MeOH/0.05% of DPPH in MeOH. Stained silica plate revealed a purple background with yellow spots at the location of the drops, which showed radical scavenger capacity. The intensity of the yellow color depends upon the amount and nature of radical scavenger present in the flavonoids.

### 2.6. 2,2-Diphenyl-1-picrylhydrazyl (DPPH) Radical Scavenging Assay

Each sample's stock solution was diluted to final concentrations of 1000–0.4889 *μ*g/mL in MeOH. A total of 1 mL of a 0.3 mM DPPH methanolic solution was added to 2.5 mL of sample solution of different concentrations and allowed to react at room temperature. After 30 min, the absorbance (Abs) values were measured at 517 nm with a microplate reader (Tecan NanoQuant Infinite M200, Switzerland) and converted into the percentage antioxidant activity using the following equation described previously [26–28]:

(1) %  antioxidant  activity=100−Abssample−Abssample  blankAbscontrol−Abscontrol  blank×100.

MeOH + compound solution was used as a blank, while DPPH solution plus MeOH was used as a negative control. The positive controls were DPPH solution plus 1 mM ascorbic acid. The IC_50_ values were calculated by linear regression of plots. The average percent of scavenging capacity was taken from three replicates.

### 2.7. ABTS^+^ Radical Scavenging Assay

An improved ABTS^+^ radical decolorization assay was carried out involving direct production of the blue/green ABTS^+^ chromophore through the reaction between ABTS and potassium persulfate. Addition of antioxidant to the preformed radical cation reduces it to ABTS to an extent on a time scale depending on the antioxidant activity, the concentration of the antioxidant, and the duration of the reaction. 1 ml ABTS was dissolved in water to make 7 mM concentration of the resultant solution. ABTS^+^ radical was produced by reacting ABTS stock solution with 2.45 mM potassium persulfate. The resultant reaction mixture was allowed to stand in the dark at room temperature for 12–16 h before being used. The ABTS^+^ radical solution was diluted with MeOH to an absorbance of 0.7 (±0.02) at 734 nm. Then, 100 *μ*l of sample at various concentrations (1000–0.4889 *μ*g/mL) was mixed with 100 *μ*L of diluted ABTS^+^ radical. Each concentration was analyzed in triplicate and the percentage decrease of absorbance at 734 nm with a microplate reader (Tecan NanoQuant Infinite M200, Switzerland) was calculated for each point and the antioxidant capacity of the tested compounds was expressed as percent inhibition (%). The radical scavenging activity of the compounds was measured according to the following equation [29]:

(2) $$\begin{array}{c} \%\;\;inhibition=\frac{A\left(control\right)-A\left(sample\right)}{A\left(control\right)}\times 100. \end{array}$$

IC_50_ values were calculated by linear regression of plots, and the average percent of scavenging capacity was taken from three replicates. Trolox was used as a standard in comparison for the determination of the antioxidant activity of the flavonoids.

### 2.8. Xanthine Oxidase Inhibitory Assay (XOI) 

XO catalyzes the oxidation of hypoxanthine to xanthine and subsequently to uric acid. The inhibition of XO reduces both vascular oxidative stress and circulating levels of uric acid. This assay is based on the fact that the superoxide anions are generated by the xanthine. The xanthine oxidase inhibitory activity was measured spectrophotometrically (Tecan NanoQuant Infinite M200, Switzerland) at 295 nm under aerobic condition with slight modification [30–32]. The reaction mixture contained 50 mM phosphate buffer (pH 7.5), 0.15 mM xanthine (substrate), and 0.3 U/mL enzymes (xanthine oxidase from buttermilk). Samples were prepared by dissolving 1 mg in 5% DMSO in MeOH which gives sample concentration of 144.0 *μ*g/mL in assay. The final concentration of MeOH in the reaction mixture did not exceed 1% (v/v), of which concentration did not influence the activity of xanthine oxidase. The absorption increment at 295 nm indicates the formation of 1 mmol of uric acid/min at 25°C. Allopurinol (5.0–100 *μ*g/mL), a known XO inhibitor [21], was used as the positive control. All determination was done in triplicate and half maximal concentration (IC_50_) values were calculated from the percentage of inhibition. XO inhibition activity was expressed as the percentage inhibition of XO as follows:

(3) $$\begin{array}{c} \%\;\;of\;\;inhibition=\frac{Abs\left[\left(A-B\right)-\left(C-D\right)\right]}{Abs\left(A-B\right)}\times 100, \end{array}$$

where *A* is the negative control with *B* as the blank of *A* which is without the enzyme and the sample while *C* is the activity of the sample with enzyme with *D* as the blank of the sample without enzyme [33].

### 2.9. Ferric Reducing Antioxidant Power (FRAP)

This assay was based on the reduction of the ferric tripyridyltriazine (Fe III-TPTZ) complex to the ferrous ion (Fe II) at a low pH forming an intense blue color. In the FRAP assay, compounds that are rich in antioxidant effect exert their action by breaking the free radical chain by donating a hydrogen atom. Briefly, ferric reducing antioxidant power (FRAP) reagent (2.5 mL of a 10 mM TPTZ solution in 40 mM HCl, 2.5 mL of 20 mM FeCl_3_, and 25 mL of 0.1 M acetate buffer pH 3.6) was prepared and incubated for 10 min at 37°C. Then 20 *μ*L of each compound and ascorbic acid (standard) and 40 *μ*L of FRAP reagent were added to 140 *μ*L of distilled water in a 96-well plate producing a blue-colored solution. The solutions were kept at room temperature for 20 min in the dark and then measured at 593 nm with a microplate reader (Tecan, Switzerland) using a reagent blank composed of 40 *μ*L of FRAP reagents in 200 *μ*L of distilled water [34]. A calibration curve was prepared with a serial dilution of ascorbic acid (standard). The total antioxidant capacity was calculated by interpolating the measured absorbance of the calibration curve and expressed as ascorbic acid equivalents. The results were corrected for dilution and expressed as AAE *μ*g of ascorbic acid/g.

### 2.10. *α*-Glucosidase Inhibitory Assay

*α*-Glucosidase inhibitory activity of the flavonoids was carried out according to the standard method with minor modification [35]. Briefly, 1 mg of each sample was dissolved in 10% of DMSO and MeOH as the stock solution, while, for the positive control, 1 mg of quercetin (Sigma-Aldrich, Singapore) was dissolved in 1 mL of DMSO. A solution of 30 mM phosphate buffer (pH 6.5) was used to prepare stock solution of the sample to form a final concentration of 160 *μ*g/mL. Then, 10 *μ*L/well of sample was mixed with 15 *μ*L/well of *α*-glucosidase from* Saccharomyces cerevisiae* (Megazyme, Ireland). 50 mM buffer (pH 6.5) was used to prepare the enzyme solution. Then, 115 *μ*L/well of 30 mM buffer (pH 6.5) was added and incubated for 5 min at room temperature. Then, 75 *μ*L/well of PNP-glucoside was added to initiate reaction and incubated at room temperature for 15 min. Finally, about 50 *μ*L glycine at pH 10 was added to stop the reaction. The final volume of 250 *μ*L of compound, positive control, enzyme, 30 mM buffer, and substrate was located in 96-well microplate. The absorbance (Abs) was measured using microplate reader (Tecan NanoQuant Infinite M200, Switzerland) at 405 nm to represent the amount of p-nitrophenol released from PNP-glucoside. The IC_50_, the sample concentration needed to inhibit 50% *α*-glucosidase, was determined using linear regression analysis. The equation given below was used to calculate the inhibitory activity (%):

(4) Inhibitory  activity%=Abs  control−Abs  sampleAbs  control×100%.

### 2.11. Dipeptidyl Peptidase IV (DPP-4) Inhibitory Assay

Dipeptidyl peptidase (IV) inhibitors work by inhibiting the action of this enzyme, thereby prolonging the activity of incretins that play an important role in insulin secretion and blood glucose control regulation. DPP-4 inhibitory assay was performed as per the standard method (Cayman, USA). Firstly, 1 mg of sample was dissolved in 10% of DMSO and MeOH as the stock solution. For initial activity (100%), 10 *μ*L of MeOH, 30 *μ*L of diluted assay buffer, and 10 *μ*L of diluted DPP-4 (Cayman, USA) were added to 96-well microplate. For positive control, 10 *μ*L of sitagliptin was added with 30 *μ*L of diluted assay buffer and 10 *μ*L of diluted DPP-4. For background well, 10 *μ*L of MeOH and 40 *μ*L of diluted assay buffer were added. Then, 10 *μ*L of prepared sample and 30 *μ*L of diluted assay buffer were added with 10 *μ*L of diluted DPP-4. After that, 50 *μ*L of substrate solution was added to all wells to initiate the reaction and further incubated in the dark at 37°C for 30 min. Finally, plate was read at 360 nm of excitation wavelength and 465 nm of emission wavelength using fluorescence microplate reader (Perkin Elmer, Germany). All determinations were done in triplicate and half maximal concentration (IC_50_) values were calculated from the percentage of inhibition based on the linear regression analysis. The equation given below was applied to calculate the inhibitory activity (%):

(5) Inhibitory  activity%=Initial  Activity−SampleInitial  Activity×100%.

### 2.12. Statistical Analysis

Data were collected and expressed as the mean ± standard error of mean (SEM) of three independent experiments and analyzed for statistical significance from each control. The data were tested for statistical differences by one-way ANOVA followed by Tukey's and Dunnett's multiple comparison tests of MiniTab (version 18). The criterion for significance was set at *p* < 0.05.

## 3. Results

### 3.1. Spectral Data of Tested Flavonoids

*Wogonin (5,7-Dihydroxy-8-methoxyflavone, Norwogonin 8-Methyl Ether)*. ^1^H-NMR [600 MHz, Acetone-d_6_, *δ* (ppm)]: 6.67 (s, 1H, H-3), 6.20 (s, 1H, H-6), 7.97 (m, 2H, H-2′/H-6′), 7.50 (m, 3H, H-3′/H4′/H5′), 3.84 (s, -OCH_3_, 3H, H-8a), 12.43 (s, 1H, OH-5) [36].

*Methyl Ether of Wogonin (5,7,8-Trimethoxyflavone)*. ^1^H-NMR [600 MHz, Acetone-d_6_, *δ* (ppm)]: 6.77 (s, 1H, H-3), 6.73 (s, 1H, H-6), 8.11 (m, 2H, H-2′/H-6′), 7.63 (m, 3H, H-3′/H4′/H5′), 3.95 (s, 2x -OCH_3_, 6H, H-7a, H-8a), 4.07 (s, -OCH_3_, 3H, H-5a) [37]. 

*Acetate of Wogonin (5,7-Diacetoxy-8-methoxyflavone)*. ^1^H-NMR [600 MHz, Acetone-d_6_, *δ* (ppm)]: 6.97 (s, 1H, H-3), 6.77 (s, 1H, H-6), 8.10 (m, 2H, H-2′/H-6′), 7.64 (m, 3H, H-3′/H4′/H5′), 4.09 (s, -OCH_3_, 3H, H-8a), 2.41 (s, -OCOCH_3_, 3H), 2.34 (s, -OCOCH_3_, 3H) [25]. 

*Techtochrysin (5-Hydroxy-7-methoxyflavone)*. ^1^H-NMR [600 MHz, MeOD-d_4_, *δ* (ppm)]: 6.43 (s, 1H, H-3), 6.34 (d, *J* = 2.4 Hz, 1H, H-6), 6.50 (d, *J* = 2.4, 1H, H-8), 7.87 (dd, *J* = 1.8, 4.2 Hz, 2H, H-2′/H-6′), 7.44 (m, 3H, H-3′/H-4′/H5′), 3.74 (s, 3H, 7-OCH_3_) [38]. 

*Chrysin (5,7-Dihydroxyflavone)*. ^1^H-NMR (600 MHz, Acetone-d_6_, *δ* (ppm)): 6.31 (d, *J* = 2.2 Hz, H-6), 6.60 (d, *J* = 2.2 Hz, H-8), 6.80 (s, H-3), 7.63 (m, H-3′,4′,5′), 8.09 (dd, *J* = 1.7 and 7.45 Hz, H-2′,6′), 12.91 (s, 1H, OH-5) [39]. 

*Norwogonin (5,7,8-Trihydroxyflavone)*. ^1^H-NMR [600 MHz, Acetone-d_6_, *δ* (ppm)]: 6.77 (s, 1H, H-3), 6.36 (s, 1H, H-6), 7.37 (m, 3H, H-3′/H4′/H5′), 8.13 (m, 2H, H-2′/H-6′), 12.34 (s, 1H, OH-5) [40]. 

*Acetate of Norwogonin (5,7,8-Triacetoxyflavone)*. ^1^H-NMR [600 MHz, Acetone-d_6_, *δ* (ppm)]: 6.98 (s, 1H, H-3), 6.85 (s, 1H, H-6), 8.00 (m, 2H, H-2′/H-6′), 7.67 (m, 3H, H-3′/H4′/H5′), 2.48 (s, -OCOCH_3_, 3H), 2.37 (s, 2x -OCOCH_3_, 6H) [25]. 

*Isoscutellarein (4*′*,5,7,8-Tetrahydroxyflavone)*. ^1^H-NMR (600 MHz, Acetone-d_6_, *δ* (ppm)): 6.26 (1H, s, H-6) 6.72 (1H, s, H-3), 6.93 (2H, d, *J* = 8.4 Hz, 3′, 5′) 8.00 (2H, d, *J* = 8.4 Hz, H-2′, 6′), 8.81 (1H, s, OH-4′), 10.44 (1H, s, OH-8), 10.59 (1H, s, OH-7), 12.36 (1H, s, OH-5) [24]. 

*Hypolaetin (3*′*,4*′*,5,7,8-Pentahydroxyflavone (8-Hydroxyluteolin))*. ^1^H-NMR (600 MHz, Acetone-d_6_, *δ* (ppm)): 6.27 (1H, s, H-6), 6.59 (1H, s, H-3), 6.90 (1H, d, *J* = 2.4 Hz, H-2′), 7.43 (1H, d, *J* = 2.4 Hz, H-5′), 7.46 (1H, dd, *J* = 2.4, 2.4 Hz, H-6′), 8.77 (1H, s, OH-4′), 9.52 (1H, s, OH-3′), 10.64 (1H, s, OH-7), 9.98 (1H, s, OH-8), 12.37 (1H, s, OH-5) [24]. 

*Kaempferol (4*′*,3,5,7-Tetrahydroxyflavone)*. ^1^H-NMR (600 MHz, Acetone-d_6_, *δ* (ppm)): 6.27 (1H, d, *J* = 1.8 Hz, H6), 6.54 (1H, d, *J* = 1.8 Hz, H-8), 7.02 (2H, dd, *J* = 2.4, 9 Hz, H-3′, 5′), 8.16 (2H, dd, *J* = 1.8, 8.4 Hz, H-2′,6′), 12.17 (1H, s, OH-5) [24]. 

*Quercetin (3,3*′*,4*′*,5,7-Pentahydroxyflavone)*. ^1^H-NMR [600 MHz, Acetone-d_6_, *δ* (ppm)]: 6.28 (d, *J* = 2.4 Hz, 1H, H-6), 6.53 (d, *J* = 1.8, 1H, H-8), 7.01 (d, *J* = 8.6 Hz, 1H, H-5′), 7.71 (dd, *J* = 2.0, 8.4 Hz, 1H, H-6′), 7.84 (d, *J* = 2.2 Hz, 1H, H-2′), 12.16 (1H, s, OH-5) [24].

### 3.2. Rapid Screening of Radical Scavenging Activity by Dot Blot Assay

**Figure 3:**
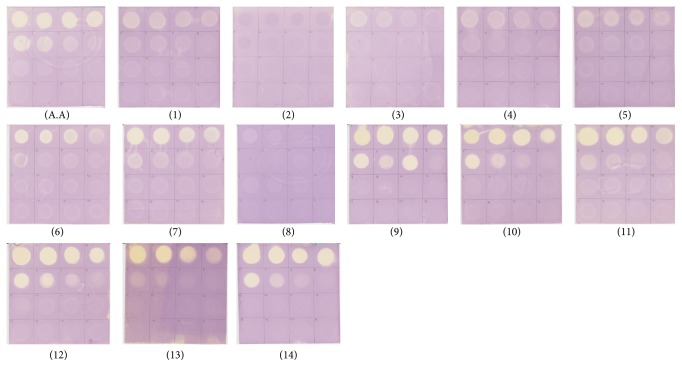
Rapid screening of radical scavenging activity by dot blot assay on a silica sheet stained with a DPPH solution in MeOH at 16 different concentrations, namely, 1000, 500, 250, 125, 62.5, 31.25, 15.63, 7.81, 3.91, 1.95, 0.97, 0.488, 0.244, 0.122, 0.06, and 0.03 *μ*g/mL applied from top to down. (A.A) ascorbic acid (positive control), (1) wogonin, (2) methyl ether (wogonin), (3) acetate (wogonin), (4) techtochrysin, (5) 8-hydroxy-7-methoxyflavone, (6) chrysin, (7) norwogonin, (8) acetate (norwogonin), (9) isoscutellarein, (10) hypolaetin, (11) kaempferol, (12) quercetin, (13) (+)-catechin, and (14) (−)-epicatechin.

For the rapid screening of radical scavenging activity, the appearance of yellow spots has a potential value for the indirect evaluation of the antioxidant of the flavonoids. When flavonoids were analyzed, more reactive flavonoids showing strong intensities of white-yellow spots appeared up to several dilutions of 1000 *μ*g/mL of the compounds. Among the groups, isoscutellarein, quercetin, hypolaetin kaempferol, (+)-epicatechin, and (+)-catechin showed strong radical scavenging activity; wogonin, norwogonin, chrysin, techtochrysin, and 8-hydroxy-7-methoxyflavone showed intermediate radical scavenging activity, while semisynthetic flavonoids, that is, methyl ether (wogonin) and acetate (wogonin and norwogonin), did not show any spot similar to the purple background as compared to the positive control, namely, ascorbic acid (Figure 3).

According to the color intensities, the overall order of the decreasing radical scavenging activity was found to be in the order of isoscutellarein > quercetin > hypolaetin > kaempferol > (−)-epicatechin > (+)-catechin > norwogonin > chrysin > wogonin > techtochrysin > 8-hydroxy-7-methoxyflavone. The result corresponded to the result of DPPH radical scavenging assay.

### 3.3. DPPH Radical Scavenging Assay

DPPH is a stable free radical that can accept an electron or hydrogen radical to become a stable diamagnetic molecule. DPPH radical reacts with suitable reducing agent producing new bond, thus changing the color of the solution. The solution loses color with the increased concentration of antioxidant as the electrons are taken up by DPPH radical from the antioxidant which can be monitored spectrophotometrically by decrease in absorbance at 517 nm [23]. Results in Table 1 demonstrate that hypolaetin expressed the lowest value of IC_50_ (3.69 ± 0.11 *μ*g/mL), statistically similar to positive control, ascorbic acid (4.75 ± 0.91 *μ*g/mL), followed by isoscutellarein, quercetin, (−)-epicatechin, kaempferol, (+)-catechin, norwogonin, and 8-hydroxy-7-methoxyflavone at 5.23 ± 0.53, 7.76 ± 0.99, 9.92 ± 0.33, 10.89 ± 0.86, 14.34 ± 1.55, 35.61 ± 1.68, and 68.24 ± 3.70 *μ*g/mL, respectively, while the rest showed weak activity whereby the IC_50_ was found to be above 100 *μ*g/ml. These results further corroborate and explain the weak spots of rapid screening dot blot of DPPH staining for the same compounds.

### 3.4. ABTS^+^ Radical Scavenging Assay

This assay was based on the ability of the flavonoids to scavenge ABTS^+^ radical. Using this method, the obtained result is presented in Table 1. Contrary to DPPH method, (+)-catechin and (−)-epicatechin showed the highest ABTS^+^ radical scavenging activity (0.62 ± 0.05 and 0.70 ± 0.08 *μ*g/mL) which is statistically significant as compared to positive control, trolox (1.76 ± 0.15), followed by hypolaetin, quercetin, norwogonin, kaempferol, isoscutellarein, 8-hydroxy-7-methoxyflavone, techtochrysin, and wogonin at 0.80 ± 0.03, 0.83 ± 0.01, 1.24 ± 0.19, 1.36 ± 0.22, 1.73 ± 0.06, 3.19 ± 0.05, 45.59 ± 4.75, and 52.65 ± 2.99 *μ*g/mL, respectively. The rest showed ABTS^+^ radical scavenging activity > 100 *μ*g/mL.

### 3.5. Xanthine Oxidase Inhibition (XOI) Assay

Xanthine oxidase (XO), the key enzyme that catalyzes the final step in the conversion of purines to uric acid, thus plays a vital role in producing hyperuricemia which eventually leads to the gout inflammation [26, 27]. The xanthine oxidase inhibition activity of 14 flavonoids was tested via* in vitro* technique assay of xanthine oxidase. The validity of the method can be observed from the IC_50_ result of the positive control used, namely, allopurinol, which showed the highest inhibition at 0.163 *μ*g/mL (Table 1). Among the 14 tested flavonoids, only kaempferol and quercetin showed high 50% of inhibition concentration (IC_50_) of xanthine oxidase which was at 8.07 *μ*g/mL and 16.36 *μ*g/mL, respectively. Isoscutellarein and chrysin showed low inhibition (>100 *μ*g/mL) of xanthine oxidase while the others were found to be inactive.

### 3.6. Ferric Reducing Antioxidant Power (FRAP) Assay

The results were expressed quantitatively in terms of the ascorbic acid equivalent (AAE) *μ*g of ascorbic acid/g. A standard curve of ascorbic acid was plotted. The data in Table 2 show that the kaempferol and isoscutellarein have the highest antioxidant capacity (265.65 ± 5.46 and 262.91 ± 4.99 AAE *μ*g of ascorbic acid/g). This value was found to be more than twofold higher (*p* < 0.05) than AAE value of ascorbic acid (114.58 AAE *μ*g of ascorbic acid/g). This was followed by hypolaetin, norwogonin, (−)-epicatechin, (+)-catechin, quercetin, and 8-hydroxy-7-methoxyflavone, with 262.91 ± 4.99, 177.37 ± 1.82, 152.14 ± 7.30, 152.07 ± 1.95, 148.12 ± 4.40, and 104.92 ± 8.29 of AAE *μ*g of ascorbic acid/g, respectively. Meanwhile, acetate of norwogonin, wogonin, chrysin, techtochrysin, acetate (wogonin), and methyl ether (wogonin) showed low ferric reducing antioxidant power with 39.63 ± 1.01, 39.15 ± 1.68, 21.38 ± 1.86, 11.49 ± 0.32, 5.06 ± 3.24, and 1.68 ± 0.18 of AAE *μ*g of ascorbic acid/g, respectively.

### 3.7. *α*-Glucosidase Inhibitory Assay

Tested on yeast *α*-glycosidase, the reaction is initiated by reacting the inhibitor (sample) with the *α*-glycosidase enzyme that competes with *ρ*-nitrophenol-*ρ*-D-glucopyranosidase for the binding site, thus releasing *ρ*-nitrophenol which was measured in the assay. It is observed that quercetin showed statistically similar value of IC_50_ to positive control; meanwhile isoscutellarein and kaempferol showed significantly higher values of IC_50_ (*p* < 0.05) in inhibiting *α*-glycosidase activity at 4.92 ± 7.06, 7.15 ± 0.96, and 12.19 ± 4.63 *μ*g/ml, respectively. Meanwhile, hypolaetin showed a good inhibitory activity at 48.42 ± 9.71 *μ*g/ml. Wogonin, acetate of wogonin, techtochrysin, 8-hydroxy-7-methoxyflavone, chrysin, and norwogonin showed high value of IC_50_ (>100 *μ*g/ml) and the rest of the compounds were found to be inactive (Table 3).

### 3.8. Dipeptidyl Peptidase IV (DPP-4) Inhibitory Assay

For DPP-4 inhibitory assay, the reaction is based on the cleavage of the peptide bond by DPP releasing the free Gly-Pro-aminomethylcoumarin group, a fluorogenic substrate resulting in fluorescence excitation and emission. The result revealed that quercetin and isoscutellarein showed the highest IC_50_ value at 21.75 ± 5.81 and 22.23 ± 1.52 *μ*g/ml which is statistically similar to positive control, sitagliptin at 24.51 ± 1.01, followed by hypolaetin and kaempferol at 34.89 ± 7.44 and 45.93 ± 8.61 *μ*g/ml, respectively. Meanwhile, wogonin, methyl ether (wogonin), techtochrysin, 8-hydroxy-7-methoxyflavone, norwogonin, and acetate (norwogonin) showed weak inhibitory activity (>100 *μ*g/ml), and the remaining compounds showed no activity (Table 3).

## 4. Discussion

**Figure 4:**
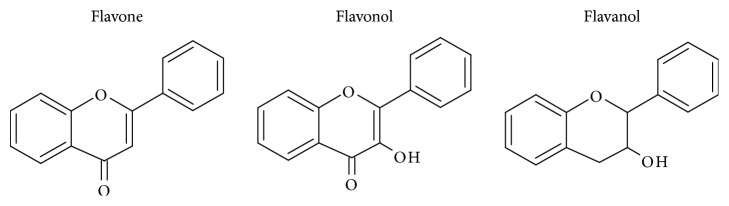
Structure of the different classes of flavonoids.

This study has compared the bioactivities of 14 different compounds comprising three structural variations of flavonoids which are flavones (absence of hydroxyl group at position 3), flavonol (presence of hydroxyl group at position 3), and flavanol (absence of double bond and ketonic group at positions 2-3 and 4, resp.) (Figure 4).

The antioxidant and radical scavenging activities of the flavonoids tested have been shown in Tables 1 and 2 which could be attributed to the high reactivity of hydroxyl substituents. For instance, quercetin, hypolaetin, (−)-epicatechin, and (+)-catechin have five hydroxyl groups attached to them which make them potent antioxidant and radical scavengers.  Table 4 depicts the chemical structures and substituents where the number and configuration of hydroxyl groups are connected to their bioactivity. However, increase of activity was also observed to depend on the configuration of hydroxylation rather than the number of hydroxyl groups. For example, for DPPH radical scavenging and FRAP assays, even though kaempferol and isoscutellarein have only four hydroxyl groups, the antioxidant capacity was shown higher than the (−)-epicatechin and (+)-catechin.

Most antioxidant studies on flavonoids have concluded that an ortho-dihydroxy (catechol) structure in B ring is important for high scavenging activity [4, 5, 41–43]. The B ring hydroxyl configuration in flavonoids has been reported to be the most significant determinant ROS scavenging because it donates hydrogen and an electron to hydroxyl, hydroperoxyl, and peroxynitrite radicals, stabilizing them and giving rise to a relatively stable flavonoids radical [44, 45]. It was also observed in this study that catechol substituent attached on B ring has imposed high activity on DPPH, ABTS^+^ radical scavenging, and FRAP antioxidant as demonstrated by quercetin, hypolaetin, isoscutellarein, (−)-epicatechin, and (+)-catechin. The presence of the hydroxyl groups in ring A and catechol structure or 4′-hydroxyl group in ring B seems to enhance the antioxidant activity in these radical assays. Cao et al. [44] concluded that a catechol moiety on ring B is essential for a high scavenging activity and this activity is proportional to the number of hydroxyl groups. The hydroxyl substituents on radical scavenging activity are considered to be the source of hydrogen atom that neutralizes radical species [46].

Alkylation and acetylation of phenolic groups at positions C-5, C-7, and C-8 (i.e., methoxy or acetate groups) were found to decrease the antioxidant and scavenging activities as observed from the result of DPPH radical scavenging assay, FRAP assay, and xanthine oxidase inhibitory assay of semisynthesized wogonin and norwogonin analogs. Studies showed that replacing the active hydroxyl groups by one or more methoxy groups significantly decreased the activity [41–43]. Moreover, in ABTS+ radical scavenging assay, methoxy group seemed to show minimal effect to support antioxidant activity as shown by wogonin, techtochrysin, and 8-hydroxy-7-methoxyflavone. All these three flavonoids have methoxy groups attached to positions C-8 and C-7, respectively, which showed minimal role of bioactivity which was also supported by the study carried out by Jang et al. [47].

For xanthine oxidase inhibitory assay, among different classes of flavonoids, only flavonol (quercetin and kaempferol) expressed significant xanthine oxidase inhibitory effect. Meanwhile, isoscutellarein, hypolaetin, chrysin, and techtochrysin were seen to demonstrate low xanthine oxidase inhibitory activity (Table 2). This observation further illustrates the importance of the C-3 hydroxyl group, C-2-C-3 double bond, and ketone group at C-4 with regard to exerting the inhibitory effect against xanthine oxidase. (+)-Catechin and (−)-epicatechin were shown to be inactive even though both compounds exhibited strong DPPH, ABTS^+^ radical scavenging activities and FRAP assay activity, which means catechol structure of B ring, which gives antioxidative potential to flavonoids, was not related to xanthine oxidase inhibition [5]. Comparing the inhibition of xanthine oxidase with that of flavanol and flavonol groups, it can be observed that the presence of hydroxyl group at C-3 and absence of 4-ketonic group and C-2-C-3 double bond reduced the xanthine oxidase inhibitory effect. Additionally, with a double bond between C-2 and C-3, ring B will be coplanar with rings A and C due to the conjugation. The saturation of this double bond will annihilate its conjugation and planarity. This implies that a planar flavonoids structure such as flavone structure is essential for xanthine oxidase inhibition as the same observation was also supported by several studies [5, 45–47].

One of the therapeutic approaches to managing diabetes mellitus is to retard the absorption of glucose via inhibition of digestive enzymes in the digestive organs such as the *α*-glucosidase [48, 49] and *α*-amylase [50]. During the last 20 years, naturally occurring flavonoids and synthetic analogs have been extensively studied as *α*-glucosidase inhibitors [51]. *α*-Glucosidase is a membrane bound enzyme located at the epithelium of the small intestine that catalyzes the cleavage of glucose from disaccharides to monosaccharides [52]. Inhibitors of *α*-glucosidase are used to control the blood sugar levels for type 2 diabetes mellitus. Usually, *α*-glucosidase inhibitors are consumed with meals as they act to decelerate the breakdown of complex sugars into glucose resulting in a delay in glucose absorption which lowers postprandial blood sugar levels [53]. This study demonstrated that quercetin from flavonol group has the highest IC_50_ value as compared to other flavanoids. A study by Xu [54] has reported that dihydroxyl groups at positions C-3′ and C-4′ (catechol) of flavonoids are effectively conjugated with the active-site residues of *α*-glucosidase. Existence of catechol system on the B ring of flavonoids is expected to contribute to the distribution of electron cloud which then becomes accessible to donate hydrogen atoms to form hydrogen bonds with active-site residues of *α*-glucosidase, thereby playing a crucial role in inhibiting its action [55]. However, isoscutellarein and kaempferol have only one hydroxyl group attached on the B ring at C-4′ position as compared to quercetin (Table 4). Due to lack of essential catechol system in both isoscutellarein and kaempferol, the electron cloud density of B ring could be slightly decreased, which may be responsible for less interaction with the binding sites of *α*-glucosidase residues and, therefore, they are expected to exert less inhibitory action; consequently, both compounds exhibited slightly less potent *α*-glucosidase inhibitory activity compared to quercetin. Owing to its potent digestive enzymes inhibitory action, quercetin has been recently recognised as positive control in many* in vitro* antidiabetic studies using *α*-glucosidase as one of the subjects to determine antidiabetic activity for natural as well as synthetic compounds [52]. In addition to that, kaempferol and isoscutellarein have also been reported as promising *α*-glucosidase inhibitors due to their potent *α*-glucosidase inhibitory activity [56, 57].

Dipeptidyl peptidase IV (DPP-4) inhibitors are one of the newest therapeutic agents against type 2 diabetes mellitus [58–61]. DPP-4 is a serine exopeptidase hormone, known to degrade two major gut incretin hormones that stimulate insulin release, that is, glucagon-like peptide-1 (GLP-1) and glucose-dependent insulinotropic polypeptide (GIP), leading to a very short half-life (approximately 2 min) of the hormones [58–61]. Inhibition of DPP-4 will prolong the half-life of GLP-1 and GIP hormones, resulting in the elevation of plasma insulin levels in human body. Result showed that quercetin from the flavonol group revealed the highest IC_50_ to inhibit DPP-4 activity, followed by isoscutellarein and hypolaetin from the flavone group. Moreover, kaempferol from the flavonol group also showed good inhibitory activity of DPP-4. These results have further highlighted the importance of hydroxyl group's presence in expressing bioactivity of flavonoids which are in line with the results from Fan et al. study [62]. Quercetin and hypolaetin have five hydroxyl groups; meanwhile isoscutellarein and kaempferol have four hydroxyl groups attached to rings A, B, and C, respectively. Additionally, the configuration of hydroxyl groups also plays an important role and the catechol/hydroxyl group attached on B ring has been found to enhance *α*-glucosidase and DPP-4 inhibitory activities. Moreover, lack of C-2-C-3 double bond and ketonic group at C-4 appeared to lower the inhibitory activity for both the *α*-glucosidase and DPP-4 antidiabetic effects.

**Figure 5:**
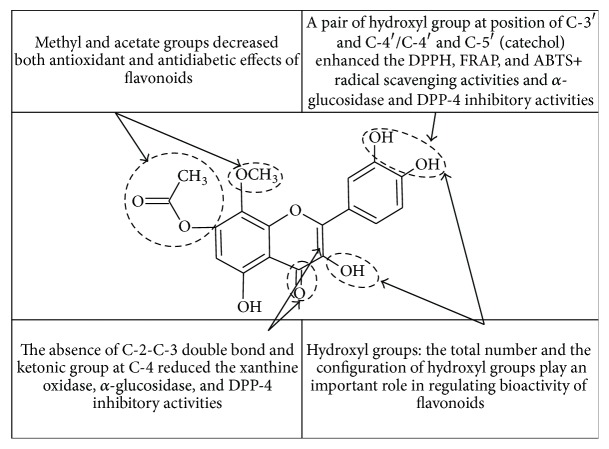
Summary of SAR study of antioxidant and antidiabetic effects of flavonoids.

To summarize, results of antioxidant and antidiabetic assays have shown that the chemical criterion is fundamental for the bioactivity of these polyphenolic compounds (Figure 5). The spatial arrangement and alkyl substitution are the remarkable determinants of antioxidant and antidiabetic activities compared to the flavan backbone alone. Also, both configuration and the total number of hydroxyl groups substantially influence the mechanisms of radical scavenging [63] and antidiabetic effects. Thus, the total of hydroxyl groups, hydroxyl configuration, C-2-C-3 double bond, and C-4 ketonic functional group are the essential features in the manifestation of bioactivity of flavonoids especially for antidiabetic effect.

## 5. Conclusion

The radical derivatives of oxygen (ROS) are the most important free radical in biological systems and harmful byproducts generated during normal cellular functions. Increasing intake of natural antioxidants may help to maintain a tolerable antioxidant status, thus preventing the oxidative stress that could lead to pathogenesis of diabetes mellitus. Flavonoids are one of the most important groups of bioactive compounds among secondary metabolites. We have reported* in vitro* antioxidant and antidiabetic activities of some selected flavonoids. This study has further revealed that the total number and configuration of hydroxyl groups existing on the compounds increased the antioxidant and antidiabetic effects of flavonoids. The absence of C-2-C-3 double bond as well as ketonic group at C-4 in ring C reduced the xanthine oxidase, *α*-glucosidase, and DPP-4 inhibitory activities. It was also discovered that the presence of catechol system on the ring B is not significant enough without the presence of C-2-C-3 double bond and ketonic group at C-4 position for the manifestation of antioxidant and antidiabetic effects. Moreover, alkylation or acetylation of hydroxyl groups on ring A decreased the bioactivity of flavonoids revealing their incapability of interacting with the binding sites of enzymes as well as quenching ROS. The results should be encouraged in future* in vivo* studies, which could ultimately lead to the development of nutritional product and semisynthetic analogs that retain substantial antidiabetic capacity with minimal adverse effects.

## Figures and Tables

**Table 1:** IC_50_ values of flavonoids for DPPH, ABTS^+^, and xanthine oxidase inhibition assays.

Samples (*µ*g/ml)	DPPH	ABTS^+^	Xanthine oxidase
Ascorbic acid	4.75 ± 0.91^GHa^	—	—
Trolox	—	1.76 ± 0.15^^FG^^	—
Allopurinol	—	—	0.16 ± 0.30^D^
Wogonin	>100^B^	52.63 ± 2.99^D^	NA
Methyl ether (wogonin)	>200^A^	>200^A^	NA
Acetate (wogonin)	>200^A^	>200^A^	NA
Techtochrysin	>100^B^	45.59 ± 4.75^E^	>100^A^
8-Hydroxy-7-methoxyflavone	68.24 ± 3.70^C^	3.19 ± 0.15^F^	NA
Chrysin	>100^B^	>100^B^	>100^A^
Norwogonin	35.61 ± 1.68^D^	1.24 ± 0.19^FG^	NA
Acetate (norwogonin)	>100^B^	78.99 ± 66.5^C^	NA
Isoscutellarein	5.23 ± 0.53^GHa^	1.73 ± 0.06^FG^	>100^A^
Hypolaetin	3.69 ± 0.11^Ha^	0.80 ± 0.03^FG^	>100^A^
Kaempferol	10.89 ± 0.86^EF^	1.36 ± 0.22^FG^	16.36 ± 0.93^B^
Quercetin	7.76 ± 0.99^FG^	0.83 ± 0.01^FG^	8.58 ± 0.72^C^
(+)-Catechin	14.34 ± 1.55^EF^	0.62 ± 0.05^G^	NA
(−)-Epicatechin	9.92 ± 0.33^F^	0.70 ± 0.08^G^	NA

Values represent mean ± SEM (*n* = 3), NA: not active; capital letters represent Tukey's test, small letters represent Dunnett's test, and means not sharing a letter are significantly different (*p* < 0.05).

**Table 2:** Result of FRAP in ascorbic acid equivalent (AAE).

Samples	FRAP (AAE *μ*g)
Ascorbic acid (positive control)	114.58 ± 0.27^Da^
Wogonin	39.15 ± 1.68^E^
Methyl-ether (wogonin)	1.68 ± 0.18^G^
Acetate (wogonin)	5.06 ± 3.24^G^
Techtochrysin	11.49 ± 0.32^F^
8-Hydroxy-7-methoxyflavone	104.92 ± 8.29^D^
Chrysin	21.38 ± 1.86^F^
Norwogonin	152.14 ± 7.30^C^
Acetate (norwogonin)	39.63 ± 1.01^E^
Isoscutellarein	262.91 ± 4.99^A^
Hypolaetin	177.37 ± 1.82^B^
Kaempferol	265.65 ± 5.46^A^
Quercetin	138.93 ± 6.22^C^
(+)-Catechin	148.12 ± 4.40^C^
(−)-Epicatechin	152.07 ± 1.95^C^

Values represent mean ± SEM (*n* = 3), NA: not active; capital letters represent Tukey's test, small letters represent Dunnett's test, and means not sharing a letter are significantly different (*p* < 0.05).

**Table 3:** IC_50_ values of flavonoids for *α*-glucosidase and DPP-4 inhibition assays.

Samples	*α*-Glucosidase (*µ*g/mg)	DPP-4 (*µ*g/mg)
Quercetin (commercial)	4.30 ± 1.06^Ea^	—
Sitagliptin	—	24.51 ± 1.01^Da^
Wogonin	>100^A^	>100^A^
Methyl ether (wogonin)	NA	>100^A^
Acetate (wogonin)	>100^A^	NA
Techtochrysin	>100^A^	>100^A^
8-Hydroxy-7-methoxyflavone	>100^A^	>100^A^
Chrysin	>100^A^	NA
Norwogonin	>100^A^	>100^A^
Acetate (norwogonin)	NA	>100^A^
Isoscutellarein	7.15 ± 0.96^D^	22.23 ± 1.52^Da^
Hypolaetin	48.42 ± 9.71^B^	34.89 ± 7.44^B^
Kaempferol	12.19 ± 4.63^C^	45.93 ± 8.61^B^
Quercetin	4.92 ± 7.06^Ea^	21.75 ± 5.81^Da^
(+)-Catechin	NA	NA
(−)-Epicatechin	NA	NA

Values represent mean ± SEM (*n* = 3), NA: not active; capital letters represent significant difference based on Tukey's test (*p* < 0.05) and small letters represent significant difference based on Dunnett's test (*p* < 0.05).

**Table 4:** Chemical structures and substituents of selected flavonoids.

Class	Compound	Chemical structure	Substituents					
			3	5	7	8	3′	4′
Flavone	Wogonin	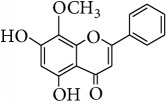	H	OH	OH	OCH_3_	H	H
	Methyl ether (wogonin)	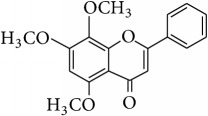	H	OCH_3_	OCH_3_	OCH_3_	H	H
	Acetate (wogonin)	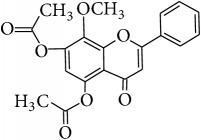	H	CH_3_COO	CH_3_COO	OCH_3_	H	H
	Techtochrysin	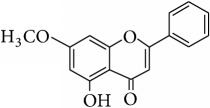	H	OH	OCH_3_	H	H	H
	8-Hydroxy-7-methoxy flavone	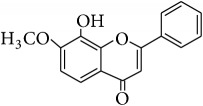	H	H	OCH_3_	OH	H	H
	Chrysin	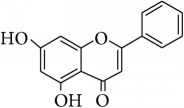	H	OH	OH	H	H	H
	Norwogonin	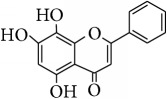	H	OH	OH	OH	H	H
	Acetate (norwogonin)	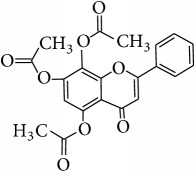	H	CH_3_COO	CH_3_COO	CH_3_COO	H	H
	Isoscutellarein	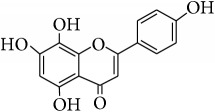	H	OH	OH	OH	H	OH
	Hypolaetin	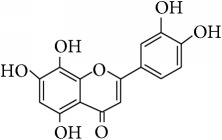	H	OH	OH	OH	OH	OH

Flavonol	Kaempferol	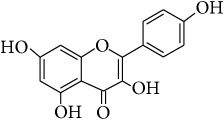	OH	OH	OH	H	H	OH
	Quercetin	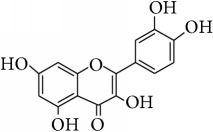	OH	OH	OH	H	OH	OH

Flavanol	(+)-Catechin (2*R*,3*S*)	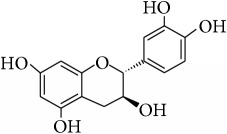	*β*-OH	OH	OH	H	OH	OH
	(−)-Epicatechin (2*R*,3*R*)	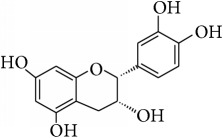	*α*-OH	OH	OH	H	OH	OH
